# *“I just want to be normal”*: A qualitative investigation of adolescents' coping goals when dealing with pain related to arthritis and the underlying parent‐adolescent personal models

**DOI:** 10.1002/pne2.12069

**Published:** 2021-12-28

**Authors:** Daniela Ghio, Rachel Calam, Rebecca Rachael Lee, Lis Cordingley, Fiona Ulph, Childhood Arthritis Prospective Study (CAPS)

**Affiliations:** ^1^ Division of Psychology & Mental Health School of Health Sciences Faculty of Biology, Medicine and Health Manchester Academic Health Science Centre University of Manchester Manchester UK; ^2^ NIHR Manchester Biomedical Research Centre Manchester Academic Health Science Centre Manchester University NHS Foundation Trust Manchester UK; ^3^ Versus Arthritis Centre for Musculoskeletal Research University of Manchester University Manchester UK; ^4^ Alder Hey Children's NHS Foundation Trust Liverpool UK

**Keywords:** adolescents, chronic and recurrent pain, coping, juvenile idiopathic arthritis, parents

## Abstract

The aim of the current study was to examine adolescents' goals when coping with pain and map these goals to the cognitive and emotional profiles of both adolescent and their parent. 17 adolescents (11‐16 years) and their parents participated in a cohort study of Juvenile Idiopathic Arthritis (JIA); the adolescents, took part in a two‐part interview (about their pain perceptions and about a recent pain experience) and the parents completed an open‐ended qualitative survey. The three datasets were analysed following a qualitative framework approach. A coping framework was developed and cognitive and emotional profiles for both adolescent and parent were mapped back to the framework. The overall goal of adolescents was to preserve social identity, by either focusing on *maintaining* a “normal” lifestyle (sub‐coping goal one) or *managing* the pain (sub‐coping goal two). Across these two sub‐coping goals, the adolescents held similar cognitive profiles (beliefs about timeline, consequences, control) but different emotional profiles such as feeling fine/happy compared with feeling angry and frustrated. Conversely, the parents' cognitive and emotional profiles were mapped back to the two groups and found that their beliefs were different across the two sub‐coping goals but had similar emotional profiles across the two groups such as worry. Both the adolescents' emotional representations and parental cognitive profiles seem to be related to how the adolescent perceives a pain event, deals with the pain, and the overall coping goal of the adolescent. Findings are suggestive that parental pain beliefs influence the adolescents' pain representations and their coping goals but are also driven by adolescents' emotions. Further work on these potential pathways is needed. Family interventions should be designed, targeting coping goals taking into consideration the importance of emotions for adolescents and parental pain beliefs.

## 1 INTRODUCTION

Juvenile idiopathic arthritis (JIA) is a relapsing‐remitting inflammatory condition presenting in children and young people, and pain is one of the main symptoms of the condition^1^ with episodic pain being highly reported.^2^ A synthesis of qualitative studies exploring young people's experiences of living with JIA found that pain is both unrelenting and unpredictable, and young people with JIA strive for normality,^3^ however, more in needed to understand how young people try to achieve normality when coping with their pain. Although JIA is described as a childhood condition it can often continue to affect individuals even into adulthood. Therefore, an understanding of how coping behaviors influence health, and supporting young people to identify coping strategies and health management techniques which minimize the impact of JIA are important for long‐term outcomes.

When examining coping (eg, the behavioral response to a stressor such as pain), there is a tendency to focus on adaptive or maladaptive coping behaviors rather than the intended goals of those behaviors. Van Damme, Crombez, and Eccleston^4^ argued that taking a motivational perspective allows us to examine coping behavior as a concept which includes the pursuit of adjustment and eventually adaptation to a long‐term condition and its features. Therefore, the goal for the coping behaviors, rather than the actual outcomes should be investigated in adolescents with JIA. Specifically, examining “why” people use their coping behaviors and “how” they cope may be useful avenues to explore further.

Chronic pain has a severe impact on health‐related quality of life and interferes with developmental changes and relationships with parents^5^ and peers.^6^ Pain in children is a family issue affecting many aspects of family functioning.^7^ Research shows that parents' experiences, beliefs, emotional well‐being, and responses to pain are associated with their child's pain outcomes,^8^, ^9^, ^10^, ^11^ and their children's engagement in activities.^12^ The parents of adolescents with higher functionality regardless of pain severity express lower levels of anxiety and stress,^9^ and parents and children are more likely to stop activities when parents catastrophize about their child's pain.^13^ The literature supports that the parents' perspective is also important to consider when examining how an adolescent copes with pain.

This paper aims to explore adolescents' motivational perspective of coping with pain related to JIA. This will be examined within the theoretical context of the common‐sense self‐regulation theory's (CS‐SRM) conceptual framework of processes involved in the initiation and maintenance of behaviors to cope with a perceived threat.^14^, ^15^ In the case of parent‐child dyads dealing with a long‐term condition such as JIA, coping could be influenced by both factors and perceptions of the child and the parent. The CS‐SRM stipulates that a patient is an active health agent and a problem solver, who develops a representation (sometimes referred to as their personal model) of their illness or health threat. These representations, both mental and emotional, are built from their experiences and their previous understanding of illness identified as illness perceptions. Five main domains of illness perceptions are thought to underpin the mental representation. These are identity: the label and symptoms associated with the illness; cause or the attributed cause of the illness; timeline: the duration of illness; consequences: the impact of the illness; control/cure: the perceived ability to control or cure the illness. To understand the interplay between beliefs, emotions, and behavioral responses within parent‐child dyads, the CS‐SRM can be helpful to look at how both agents' emotional and mental representation of an illness or symptom relates to the motivation to behavior.^14^, ^16^ Therefore, the aim of this qualitative work is to explore the coping goals and related personal models (cognitive and emotional profiles) of JIA of both the adolescents and their parent.

## 2 METHODS

### 2.1 Design

This was a qualitative cross‐sectional study. The adolescents participated in a two‐part interview, involving a computer‐assisted interview about pain experience and a cognitive interview using the Revised Illness Perception Questionnaire (IPQ‐R).^17^ The order of the two parts of the interview was randomly ordered for every adolescent using a randomization matrix. The parents were given a qualitative survey.^18^ Three phases of analyses were conducted as described below using Framework Method Analysis.^19^ The data collected from the computer‐assisted interview were organized according to Motivational Perspective,^4^ to explore “why” and “how” the young people were coping with pain. When applying an overarching theoretical approach to this data, we used the dual‐process framework proposed to understand how the “self” negotiates pursuing and adjusting goals^20^ (Phase 1). The illness perceptions data were organized according to the domains of common‐sense self‐regulation theory's (CS‐SRM) as a theoretical approach to develop personal models of both the child and the parent (Phase 2). The researchers took a contextualistic critical realist perspective when applying the frameworks and triangulating in Phase 3. The interviewer (DG) female, a student at the time of the interviews, had formal training in listening to children and young people, and working with children and young people and was supervised by experienced qualitative researchers (FU, LC, RC) who were part of the iterative discussions.

### 2.2 Participants and recruitment

For this study, adolescents with JIA and their parent/carer were approached and recruited over a period of 6 months. This data collection occurred and was completed prior to COVID‐19 pandemic. Potential participants were identified through an on‐going inception cohort study of children and adolescents presenting with new‐onset inflammatory arthritis (The Childhood Arthritis Prospective Study; CAPS).^21^ Recruitment study information packs were sent to all families of adolescents aged between 11 and 16 enrolled in CAPS at Alder Hey Hospital in Liverpool. Within the packs were reply slips to send back if they were interested in being contacted to arrange the interview before or after their child's next appointment. The UK Northwest Multicentre Research Ethics Committee approved the study.

Consent for this study was received from 25 participants. All participants gave written informed consent and assent (where appropriate) prior to interview. Due to the nature of clinic appointments, some participants could not attend the interviews, so the final sample consisted of 20 adolescents. Out of the 20, 17 parents/carers (15 mothers and two fathers) filled out the questionnaire; consequently, analysis was conducted for 17 parent/carer‐child dyads. Demographic information can be found in Table 1. Most of the adolescents were female (59%) and were aged 11‐12 (47%) most had JIA between 3 and 7 years (70%) and most were oligoarthritis‐persistent JIA category (47%). Demographic details, for example, age, were not collected for the parent/carer.

**TABLE 1:** Sample demographics from both datasets

	Frequency	*n* (%)
Adolescents		
Sex		
Male	7	(41)
Female	10	(59)
Age (years)		
11‐12	8	(47)
13‐14	4	(24)
15‐16	5	(29)
Duration of condition (years)		
All life (diagnosis before first birthday)	0	(0)
8–11 years (diagnosis in toddler years)	3	(18)
3–7 years (diagnosis in middle childhood)	12	(70)
Up to 2 year (diagnosis in adolescence)	2	(12)
JIA Category		
1. Systemic arthritis	3	(18)
2. Oligoarthritis—persistent	8	(47)
3. Oligoarthritis—extended	2	(11)
4. Polyarthritis—RF negative	3	(18)
5. Polyarthritis—RF positive	1	(6)
Parent/Carer		
Mother	15	(88)
Father	2	(12)
Other main caregiver	0	(0)

### 2.3 Materials

#### 2.3.1 Pain—in my shoes

**FIGURE 1:**
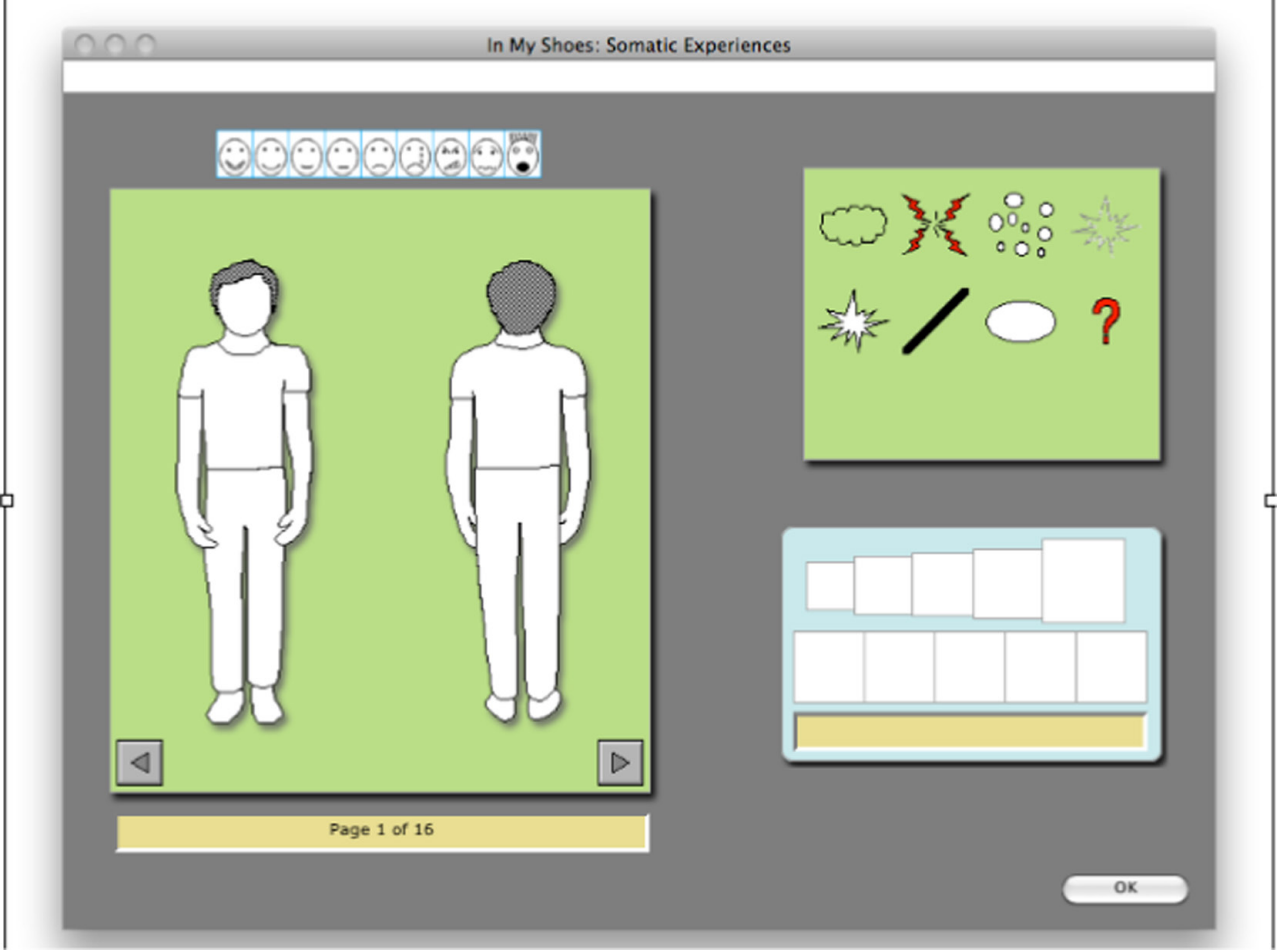
Screenshot of “In My Shoes” somatic experiences module

In My Shoes (IMS) is a computer‐assisted module interview tool.^22^, ^23^ This tool is being developed as a communication and assessment tool that consists of a number of modules, one of which is designed to assist children in describing somatic experiences (Figure 1). IMS aids adolescents in talking about pain. By allowing the adolescent to lead the descriptions of pain, the interviewer (DG) was able to use the same language to shape the questions about coping.

#### 2.3.2 Revised illness perception questionnaire (IPQ‐R) cognitive interview

The IPQ‐R^17^ assesses the five main cognitions that have been found to underpin mental representation in addition to the emotional representation from the CS‐SRM. The items of the questionnaire were used as part of the cognitive interview—further information on this procedure is provided below.

#### 2.3.3 Qualitative survey for parent/carer

While the adolescents took part in their interviews, their parent/carer completed a 2‐part qualitative survey consisting of 13 open‐ended questions. Part one contained open‐ended questions adapted from the Brief Illness Perception Questionnaire (BIPQ)^24^ to assess parents'/carers' beliefs about their child's JIA. Part two consisted of 7 open‐ended items on communicating with their child about pain, description of the last pain episode and their child's coping, as well as an account of their coping advice.

#### 2.3.4 Clinical information

Clinical information was available for each of the adolescents, and this included disease duration and the Childhood Health Assessment Questionnaire (CHAQ),^25^ a 24‐item disability index covering function, mobility, and limitations plus a 100 mm visual analog scale for pain intensity.

### 2.4 Procedure

Interviews took place in a private room in the clinic, audio‐recorded, and transcribed verbatim, on average the interviews took 58 minutes ranging from 43 to 76 minutes long. During this time, their parent/carer completed the qualitative survey for parent/carer choosing either to stay with their child or in a separate room. Names have been replaced by pseudonyms. After the interviews, the interviewer (DG) would take notes reflecting on the interview.

In My Shoes was used as a tool to aid discussion and the interview of a recent pain experience and coping with that pain. The adolescents completed three modules of IMS, the introductory module (providing demographic information), a module in which they completed an emotions panel with their own descriptors of emotions to be used to complete the somatic experiences' module. The interviewer used the adolescents' words and descriptors to probe further, exploring how the young person coped with the pain they described. Questions also included dealing with the emotion they chose for the pain. Cognitive interviewing was utilized to map the adolescents' thoughts and reasons when answering the items of the IPQ‐R.^26^ For the completion of the cognitive interviews, the two techniques of think aloud and verbal probing questions were used. The adolescents were asked to practice thinking aloud before commencing their interviews. Verbal probing was used to encourage adolescents to continue with their thinking aloud. While the interviews were structured around the questionnaire items, the think aloud method allowed exploration of the reasoning and thought process when asked each item, while the verbal probing technique allowed exploration of certain thoughts and concepts and freedom to add further questions to the interview of concepts that were coming out of the initial analysis.

### 2.5 Data analysis

The research team had three data sources to analyze qualitatively: the pain data from the IMS interviews and the illness perceptions data for the adolescents and their parents/carers. These datasets need to be analyzed separately and then combined to build an understanding of the relationships between parent‐child dyads and coping with pain. Framework analysis^19^ was ideal due to its flexibility of using both *deductive* and *inductive* approaches to complex data. There were three overarching phases to combine the pain data with the illness perceptions data. The first phase was an analysis to develop a pain coping framework that identifies the coping intent of the coping behaviors discussed by the adolescents. The next two analysis phases included developing and charting the illness perceptions datasets according to the CS‐SRM framework and then applying the coping framework to the illness perceptions datasets. This helped develop “profiles” from the adolescents' illness perceptions data and their parent's illness perception data. DG transcribed all the interviews; this allowed *immersion* and *familiarization* of all three datasets.

#### 2.5.1 Phase 1—pain dataset—to develop a coping framework

The pain data from the IMS interviews (about adolescent's pain coping) were the first dataset analyzed. This dataset's transcripts were *indexed* and summarized into a coping‐based framework. In the first charting stage, summary data of coping behaviors, intention, and coping strategies were charted and coded accordingly as part of the *detection* stage of the Framework approach. The coping behaviors and strategies were given initial categories and further indexing. The coding team, DG, LC, and FU agreed on the different categories of coping behaviors and discussed iterations of the coping framework.

*Categorization* of data in the second charting stage allowed the coding team to identify two sub‐categories for coping intent. The last stage generated the f*inal thematic framework* to be used to classify the other datasets. The final stage of the framework analysis is *mapping and interpretation* to identify what Ritchie and Spencer^19^ described as the parent theme. For this analysis, the team referred to this aspect as the superordinate theme for the coping goals. At this stage, the coding team examined the overarching categories for subordinate themes. To develop the coping framework the team used a deductive approach, starting from behaviors and working up the framework. To provide names in the framework that best captured what we were synthesizing from the pain data, we used terms from the dual‐process framework^20^ which matched with what we were developing from the data.

**FIGURE 2:**
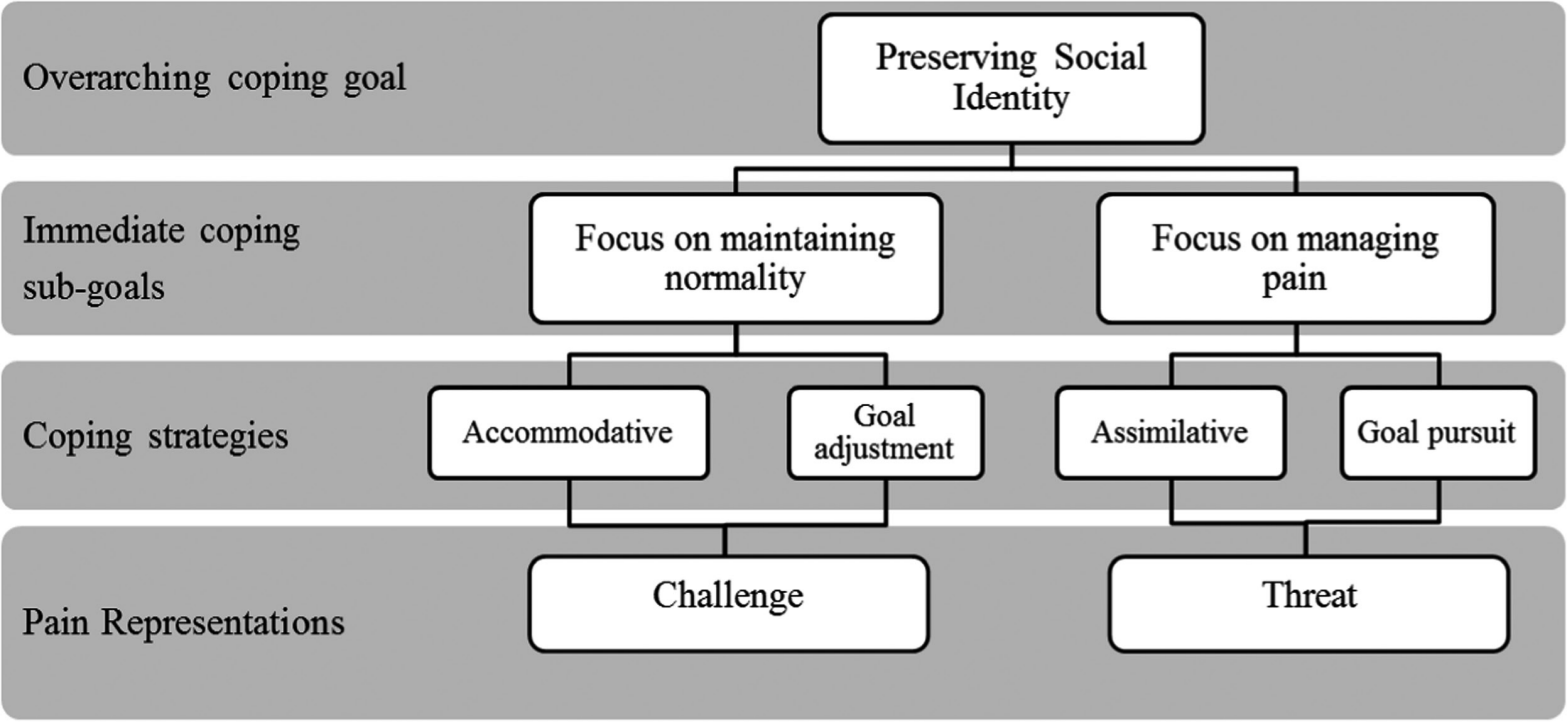
Coping framework with subordinate goal and immediate coping sub‐goals

The coping framework has three hierarchal parts (Figure 2), at the top the major theme being the higher level of coping goal that can be highly abstract ideals^27^ the second part of the framework is the lower‐level goals which can be more concrete.^27^ The bottom part of the coping framework includes the coping behaviors (that fell under four categories; physical activity, seeking support, seeking medical care, and pain disclosure).

#### 2.5.2 Phase 2—illness perception datasets—to organize individual's personal models

For the illness representations dataset, an deductive approach of the framework analysis was taken due to using a preexisting framework outlined by the Common Sense Self‐Regulatory Model (CS‐SRM).^15^ Similar stages as described above of *familiarization* and *immersion, index, charting*, *detection, categorization* and *mapping and interpreting* were used to approach the illness perceptions datasets. These stages were completed separately for the adolescents' dataset and the parents' dataset. Data management involved organizing the transcripts according to the domains and concepts of the CS‐SRM. Only the themes of timeline (both cyclical and chronicity) consequences, control/cure (both personal control and treatment control), and emotional representations were kept for this analysis. The data were subjected to further charting within these themes (six constructs) and sub‐themes were developed.

#### 2.5.3 Phase 3—combining datasets: applying coping framework developed in phase 1

**FIGURE 3:**
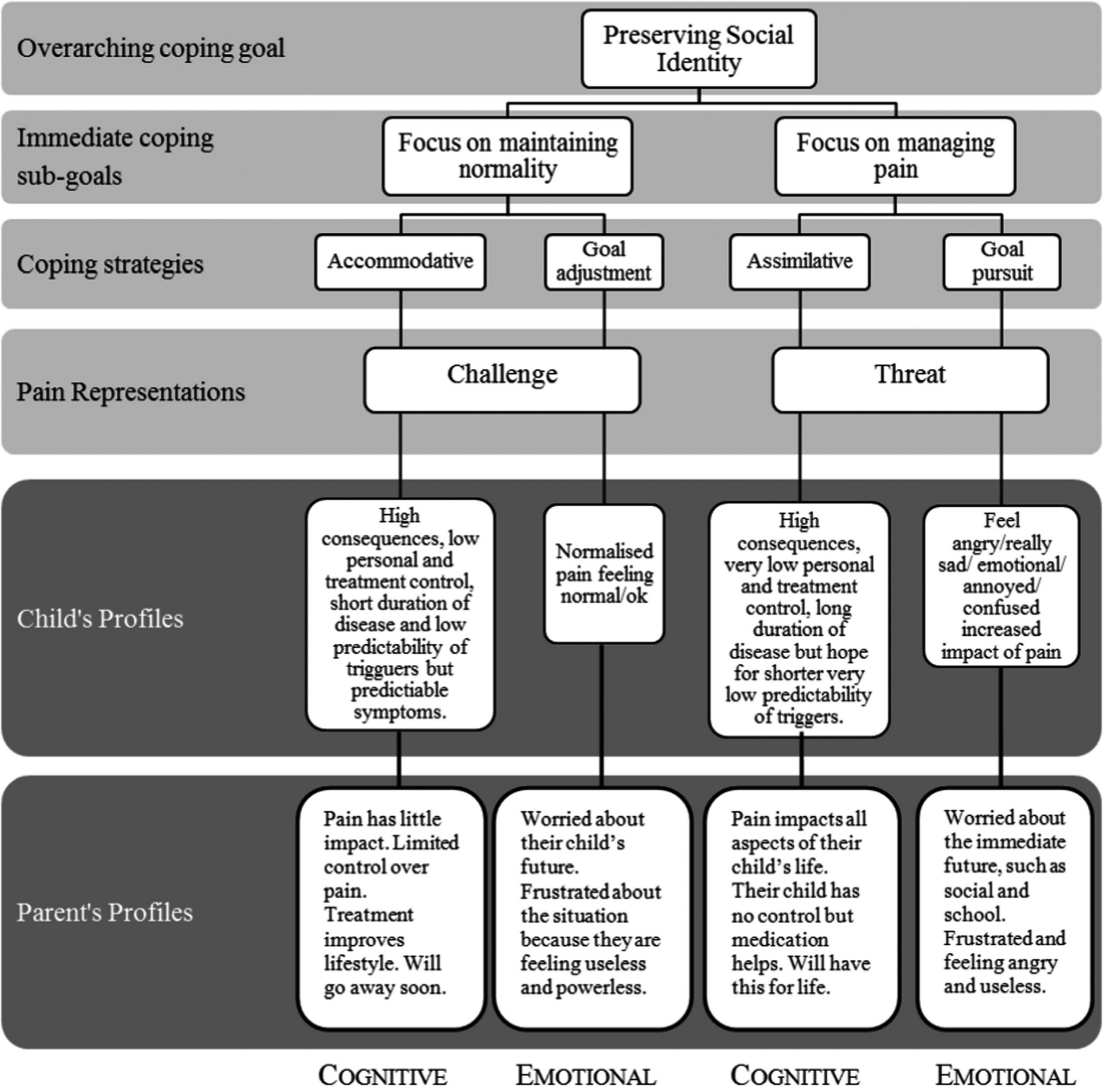
Coping framework with the cognitive and emotional profiles of the adolescents and their parents cognitive and emotional profiles

The illness perceptions datasets were organized according to the final thematic framework identified and developed from the pain dataset. Data from both the adolescent and the parent were charted and synthesized into the categories to which they were classified during the pain dataset analysis. Once the illness perception data were organized according to their classifications, there was further indexing and detection. The adolescent and parent data were examined separately. The sub‐themes previously identified during the illness perceptions dataset phase created cognitive and emotional profiles unique to the groups. Profiles were synthesized to discern the key characteristics underpinning the bottom and the middle stages of the hierarchical coping framework described in phase 1. The cognitive and emotional profiles created a unique pain representation that underlay the coping strategies and coping goals of the adolescents and their parent. This final phase permitted a comparison of the parent‐child dyads across the two coping goal categories (Figure 3).

## 3 RESULTS

The results for this paper have been organized into two sections: (a) describing the coping framework developed from the coping behaviors and strategies described by the adolescents in the IMS pain dataset interviews and then (b) applying this framework to illness perceptions datasets of the adolescents and their parents/carers.

### 3.1 Phase 1—creating the coping framework

Three categories containing a total of nine coping behaviors were identified: Physical activity, either (a) resting and relaxing (b) staying still, (c) physical activity (d) get on with it: Pain disclosure, either (e) not telling at all, (f) telling mum but not school or friends (g) telling anyone: Treatment seeking from (h) health professionals or (i) taking medication.

Using a coping motivational approach to the behaviors identified, the coping framework was created (Figure 2). The coping framework was developed through the theoretical hypothesis that actions and action plans are organized through personal motives or reasons, the *why* question would provide higher‐level goals which can be highly abstract ideals, whereas the *how* question would provide lower‐level goals which can be more concrete in the methodology. An overall subordinate theme (higher‐level goal) emerged from the pain dataset analysis; adolescent's motivation to cope was driven by wanting to live a “*normal life*.” The coping behaviors identified in the data were all working toward preserving a social identity. These adolescents recognized that pain impacted their everyday life and their self‐concept in comparison with other adolescents their age. Therefore, these adolescents have established *normality* by comparing their own functioning with what other adolescents can do. Their coping was motivated by a need to be socially perceived as others in their age groups. These coping behaviors are listed in Table 2, and Table 3 with examples of the coping intent.

**TABLE 2:** Coping categories and coping intent with an example from the adolescents who perceived pain as a challenge

Coping category	Mental process	Physical activity			Seeking support			Seek medical care	
Coping Strategy	Get on with it	Slow down or stop the activities	Rest and relax	Exercise	Do not tell anyone	Tell mum but not school	Tell anyone	Hospital	Take medication
Function/coping intent	This was a mental process coping strategy to maintain the normalization of the pain. Using this strategy, the normal standards have been adapted.	Still attempting to do activities however slower or else less of. They recognize that pain can be limiting their activities. They are comparing themselves to their usual self.	When the pain was too overwhelming to manage this strategy allowed to ease the pain back to a manageable level.	Meant making some changes (such as swapping football to swimming) it meant that they could still exercise just in a low‐impact sport.	Did not tell because of the chance of being viewed or treated differently from their peers. This would interfere with their maintaining an image and a sense of normality in their lives.	Not tell people at school because this would interfere with carrying on as a normal student like their peers. Mums understood the pain and kept tabs.	Telling anyone about their pain meant that they are understood and not judged by the people around them.	N/A	Medication is viewed as a method to aid carrying on as normal. It helps manage the pain to continue with activities.
Example	“I just carried on as normal” Kevin, age: 11	*Get on with it, try not to think about it and if it's really sore put something on it that helps like cool it down or stop it from feeling as much pain*. Daisy, age: 14	“I just tried not to move it, tried to keep it still. I did not do much because I was in pain. I couldn't do many things because I was in pain.” Gwen, age: 12	The worst is when I try to do sports, but good thing is swimming that's the thing they told us to do is swimming because you are weightless.” Adam, age: 13	“Well, I didn't want to be treated like sort of erm I don't know make a big deal about it 'cause they would probably say do you want someone to write for you and I didn't really want anyone to write for me.” Daisy, age: 14	“I don't if I get pain at school, I won't tell anyone because I just get on with it, but I don't usually get pain often at school because I am either sat down in lessons or at break and break doesn't last long so I don't walk round long to get pain”. Ian, age 15	“Talking about it makes other people understand, so I do tell a lot of people when I am in pain so that they know that I am in pain, so that they don't think I am ignoring them or not talking to them as much.” Carrie, age: 12		“Take a tablet and then it normally dies down.” Harrison, age: 16

**TABLE 3:** Coping categories and coping intent with an example from the adolescents who perceived pain as a threat

Coping category	Mental process	Physical activity			Seeking support			Seek medical care	
Coping Strategy	Ignore existence of pain	Stop Activities / Stay still	Rest and relax	Exercise	Do not tell anyone	Tell mum but not school	Tell anyone	Hospital	Take medication
Function/ coping intent	The mental process of ignoring existence of pain meant to avoid thinking about the pain. A separate entity to ignore. Especially when pain‐free they ignored it – to feel normal.	Stop doing activities when experiencing a pain episode. Measure ability by comparing peers' ability.	Resting and relaxing was a strategy to stay comfortable while waiting for the pain episode to pass.	N/A	Telling someone meant having to deal with the pain risking being perceived as different from peers and as someone unable to cope with the pain.	Telling others at school would risk not being believed and be singled out as different. Parents would believe them and would legitimize their pain episode.	N/A	Hospitals are viewed as a last resort for dealing with the pain. Seeking doctor's care happens when other routes do not alleviate the pain.	Medication is viewed as a way of helping control the pain, but they still need to wait for the pain episode to pass.
Example	“I just don't like to think about it I like to get on with it” Taya, age: 15	“I also had P E and it had started to hurt then as well so I couldn't run as fast as everyone. I just had to stop and just say that I couldn't like do it, so I didn't go as far as everyone else” Bianca, age: 11	I don't move I get comfortable and just stay there. Mia, age: 16		“I don't know I just don't like to complain so I just keep it to myself” Taya, age: 15	“I don't tell any of my friends I only tell my mum I am just scared they will like in case they think I'm attention seeker just I don't like talking to them about it because I don't like it, so I just don't talk.” Eleanor, age: 14		“If it's really bad I go back to the doctors and see if they can do anything.” Eleanor, age: 14	“it's alright taking medicine, but it doesn't stop the pain” Neil, age: 11

As shown in Figure 2, to preserve their social identity adolescents engaged in two types of coping strategies. Which coping strategy and immediate sub‐coping goal the adolescents engaged in was dependent on how they perceived their pain. The evidence of these two groups is presented below. If the adolescents perceived their pain as a challenge to their goal of being perceived normal, they adjusted their goal and were accommodative. In this group, the eight adolescents focused on continuing their everyday activities to *maintain their sense of normality* (maintenance). For the adolescents who viewed their pain as a threat to that sense of normality, they were assimilative and pursued their goal of being normal. These nine adolescents focused explicitly on *managing the pain* (management). The two sub‐coping goals will be referred to as the *maintenance* category and the *management* category.

In the maintenance category, the adolescents utilized their coping behaviors and strategized to continue with their everyday activities even if that meant limiting the amount of the activity. There were different strategies that the adolescents employed to not be treated differently from other adolescents their age, or to be perceived differently from what they would consider as the norm. For some adolescents this meant they do not tell others about their pain so that they are not given special treatment.

“I just try to ignore it. Well I didn't want to be treated, like sort of erm, I don't know make a big deal about it because they would probably say do you want someone to write for you and I didn't really want anyone to write for me.”

Daisy, age 14 (maintenance category)

Other adolescents in the *maintenance* category found that pain can affect their mood swings and their attitudes. Therefore, to preserve their social identity they explicitly tell others about their pain. This behavior was identified as an adaptation of the adolescents' norm.

“Talking about it makes other people understand, so I do tell a lot of people when I am in pain so that they know that I am in pain, so that they don't think I am ignoring them or not talking to them as much.”

Carrie, age 12 (maintenance category)

Adolescents that described using exercise to cope with the pain were only identified in the *maintenance* category. Coping intent was to continue moving their joints even if this meant changing the types of exercise they did. *“The worst is when I try to do sports, but good thing is swimming that's the thing they told us to do is swimming because you are weightless.”* Adam, age 13 (maintenance category).

The management category was derived from the accounts of the adolescents that have a coping intent to focus and manage their pain. The central driving mechanism of managing pain was by limiting function by either reducing or stopping activity when in pain. These adolescents managed their pain by adjusting or changing their plans. The focus of their coping was the pain.

“It was quite hard because I also had PE and it had started to hurt then as well so I couldn't run as fast as everyone. I just had to stop and just say that I couldn't like do it, so I didn't go as far as everyone else”

Bianca, age 11 (management category)

For some of these adolescents, the pain rather than the activity was the priority. These adolescents used pain medication to manage the pain while waiting for a pain episode to pass.

“It stopped me from doing things that I would have liked like my cousins were going into the pool and I was like I am too sore to. And I couldn't do what I would have liked to do. I took a lot of medication to make the pain leave.”

Eleanor, age 14 (management category)

These adolescents believed that waiting for the pain to pass would enable them to keep the social perception of normality in other moments. Some felt that “*Nothing will stop the pain*” (Vincent, age 12 management category) but waiting for pain to pass meant potentially experiencing a pain‐free subsequent day.

There was no difference in the mean age between the *maintenance* category (13.1 years) and the *management* category (13.4 years). The 7 boys were split across the groups (4 in the *maintenance* and 3 in the *management* category groups). The mean disease duration in the *maintenance* category was 4.0 years and 4.7 years in the *management* category. The adolescents in the *management* category reported higher mean CHAQ and pain scores (0.9 and 31.9, respectively) than those in the *maintenance* category (0.4 and 19.1, respectively).

### 3.2 Phase 2—applying the framework

When the coping framework was applied to the adolescent and parent illness perceptions datasets, there were trends and patterns in their cognitive and emotional representations of pain (Figure 3). When comparing the trends and patterns in the two coping goal categories there were distinct differences in the parents' cognitive profiles and the adolescents' emotional profiles. However, the parents' emotional profiles and the adolescents' cognitive profiles were similar across the two coping goal categories.

What follows is a presentation of the data in each cognitive and emotional pathway of the parents and their children. The next section reports the differences in the parents' cognitive profiles and the similarities in the parents' emotional profiles across their children's coping categories. That is followed by a section reporting the differences in the adolescents' emotional profiles and the similarities in the cognitive profiles.

### 3.3 Parents' cognitive profile

As highlighted above, unlike the adolescents, different patterns were found in the cognitive profiles of the parents and not the emotional profiles. In comparing the two groups of parents of children in either the *maintenance* or *management* category there were similar trends in the emotional profiles but there were different cognitive profiles.

All parents acknowledged that their child's pain affected aspects of their child's life, limiting their child's activities and exercises. However, it was only in the *maintenance* category that there were parents who believed that pain and arthritis had little impact on their child's life. *“If she has a flare up of arthritis, she realises that she can't do certain things. (Faye) has learnt to do many things and arthritis doesn't stop her.”* Faye's Mother (Faye's age 11).

Parents in the *management* category emphasized the impact pain has on their child's education and school attendance. *“All aspects, he misses a lot of school, so he is very behind, has no friends, his social life is non‐existent.”* Vincent's Mother (Vincent's age 12).

There were differences in timeline beliefs. Parents in the *management* category strongly believed their child would continue experiencing symptoms for a long time and some even believed their child's arthritis is “*For life as far as I know.”* Mia's Mother (Mia's age 16). Whereas parents in the *maintenance* category held more optimistic beliefs about the duration of their child's pain. They reported believing their child would no longer experience pain in their adulthood, hoping that their child would grow out of the condition. *“Don't know how long it will last. I'm hoping he will grow out of it.”* Ian's Mother (Ian's age 15).

Parents that believed their child had no personal control were all in the *management* category. Although the parents believed their child had some limited treatment control over the condition. *“I don't think (Lucy) has any control over her symptoms*. *I see how much the medication is helping."* Lucy's Mother (Lucy's age 11). The parents in the *maintenance* category recognized that their child may not have full control over their pain. They acknowledged there was limited control through exercise and mobility and tried to have a positive mind‐set. They also believed that treatment helped control their child's condition. They believed that treatment improved their child's lifestyle. “T*he methotrexate has given (Carrie) a better quality of life.”* Carrie's Mother (Carrie's age 12).

### 3.4 Parents' emotional profiles

Both groups of parents expressed feeling worried. In the *maintenance,* category parents expressed worry about their child's future, specifically about treatment side effects and their child's career prospects. In the *management* category, parents expressed short‐term worries. For example, worrying about the activities their child cannot take part in or their education. The worry led to some parents expressing guilt regarding their other children.

“There is an underlying worry that it's going to carry on into adulthood and may impact his future career prospects.”

Harrison's Father (Harrison's age 16, maintenance category)

“She is always our main concern how is she feeling, is she in pain today? I feel sorry for her, I think about it all the time. I feel sorry for my son as he has to fit in around (Paige) in some ways, I feel guilty, and I always try to be positive and hope both my children are happy and content and try to carry as normal as possible.”

Paige's Mother (Paige's age 15, management category)

In both categories, the parents reported feeling powerless and useless in their role. For those parents in the *management* category, they also expressed that this inability led to the parents reporting feelings of frustration, anger, or sadness.

“Useless, I can give pain relief and encourage movement so she doesn't get stiff but can't take it away fully.”

Daisy's Mother (Daisy's age 13, maintenance category)

“Sad really. Wish could do more to help her.”

Bianca's Mother (Bianca's age 11, management category)

### 3.5 Adolescents' emotional profile

When comparing the emotional profiles of the adolescents between the two coping goals groups, there were differences in the emotions identified as well as the underpinning drivers of those emotions. In both categories, there were adolescents that labeled their feelings as “angry,” “sad,” or “upset” with different underlying reasons. In the *maintenance* category, the adolescents experienced these emotions when they felt that the pain was interfering with their activities and counteracting their preservation of normality. Therefore, they had to adjust their lives. *“Arthritis is trying to bring me down”* Carrie, age 12.

Despite feeling anger these adolescents in the *maintenance* category also reported feeling fine because they could tolerate the pain and it was less likely to interfere with their life or their plans. Tolerating the pain would allow the adolescents to work toward maintaining their sense of normality. It was only in the *maintenance* category that there were adolescents that described feelings as “ok,” “fine,” or *“Normal because it is always there”* Harrison, age 16. By emotionally normalizing the pain, the adolescents were able to continue with activities and life to reach their maintenance coping goal.

In contrast, adolescents in the *management* category reported feeling “angry,” “really sad,” and feeling “emotional” “annoyed” or “confused” because they had to stop activities completely to attend to the pain. The emotions expressed by these adolescents reflected the fact that they defined their pain as episodic and dealt each pain episodes as they occurred. *“Sometimes, like in the summer, if you get stuck and you can't walk properly and being inside it's upsetting because I'm thinking I should be there or I should be there”* Zahra, age 14. Some adolescents reported anger toward the pain because of the impact it has on their lives. *“It does make me angry if it's painful and I can't play out”* Vincent, age 12.

The emotional profiles were unique to the coping category and reflected how the adolescents perceived their pain. For the adolescents in the *maintenance* category, normalizing their pain meant that pain became their norm. For the adolescents in the *management* category, normal was the period without any pain. They aimed to manage the pain episodes that were interfering with their normal.

### 3.6 Adolescents' cognitive profile

The adolescents' cognitive profiles in the two categories were similar although there were some trends underlying the beliefs that were unique to each category. This section will highlight those unique trends within very similar cognitive profiles. Adolescents in both categories believed that their pain was impacting their lives. Their beliefs about the pain consequences were driven from their experiences of limiting activities or having to decrease activities because of the pain. All adolescents recognized that their pain impacts their family's lives and may cause obstacles within their family.

“In PE, sometimes I have to sit out and I can't do many things I could do before. I still can do everything, just not as much as I used to before.

They [the family] have to do certain things for me like I can't eat with my fork, so we have to get special things for me to use”

Gwen, age 11 (maintenance category)

“I can't do certain things when it's bad [...] my sister always undresses me and things and helps me have a bath. And my mum has to do certain things for me. And this stops them from doing things they want to do.”

Mia, age 16 (management category)

All the adolescents described their pain as unpredictable, and they were unable to identify the triggers of the pain. It was only in the *maintenance* category that the adolescents added that they knew what to expect when in pain and this meant they felt prepared despite the unpredictable nature of their pain. This suggests that despite both groups of adolescents believing that their pain was unpredictable they focused on different aspects of the pain episodes. Adolescents in the *management* category focused on when the pain episode occurs, while adolescents in the *maintenance* category focused on what happened when the pain episode occurred.

“It changes from days so like one week it would be gone by the day. It's normally just the same; it's always there but it's worse on some days than others. If I wake and it's really hurting and then I wake up and it's fine.”

Harrison, age 16 (maintenance category)

“Some days I will be fine and the next I can be in pain. In quite a lot of pain and then like it can go on for weeks when I am fine but then it can appear out of nowhere.”

Bianca, age 11 (management category)

Adolescents in both groups thought they would be free of pain. The *maintenance* category adolescents are expected to continue getting better and soon be completely free of pain. This expectancy was driven from the adolescents' experiences of getting better and eventually being free of pain. *“The way that I'm actually going I think it's going to go away soon. From what I've seen so far because it's progressed a lot from what it was.”* Adam, age 13.

However, adolescents in the *management* category hoped they would grow out of it and that they would stop experiencing pain. This belief appeared to be based on the following two reasons provided in the interviews. The first reason was adolescents being told by doctors they would grow out of pain. The second relates to the name of the condition. *“It's called juvenile arthritis so I don't think it will affect me forever.”* Wyatt, Age 13.

All the adolescents perceived themselves as lacking complete personal control over pain. In both coping categories, all adolescents believed that taking medication was a form of eliciting a sense of personal control over the pain and arthritis, albeit with limitations because the treatment is not a cure. The struggle to gain personal control was evident in the adolescents; they believed that by knowing their own limits in combination of taking medication, they are gaining a level of control.

“I think that if I do a lot of exercise and keep my joints going it will get less stiff. Like I can control it, but sometimes I can't control it. It's just different. Sometimes when I run, I get tired out quickly and I don't want to be tired out because I want to do lots of exercise, so I want to keep going. Sometimes I can keep going because I just go.”

Faye, age 11 (maintenance category)

## 4 DISCUSSION

In the coping framework developed from the coping behaviors previously identified, one overarching coping goal emerged. This overarching goal of the coping framework was that the adolescents tried to preserve their social identity, namely, to be perceived as “normal.” Normality was viewed as what their peers did in everyday life. Striving for normality, as defined by their peers, is evident in previous qualitative literature.^3^, ^28^, ^29^ The current study adds a new dimension to earlier literature by demonstrating how they aimed to achieve this goal; namely via two immediate sub‐coping goal categories labeled *maintenance* category and *management* category.

These categories describe the function underlying the coping strategies of the adolescents, and which sub‐goal an adolescent tried to achieve was determined by their pain representations. In the *maintenance* category, the adolescents attempted to maintain a sense of normality by decreasing activities. The adolescents in the *management* category focused and attended to the pain to continue with normal life when the pain episode is over. The cognitive and emotional profiles were synthesized for both categories.

What emerged from the analysis was that there were no clear differences in the cognitive profiles in both categories for the adolescents. However, the emotional profiles were different. An opposite pattern was found in the parent's profiles where emotional profiles were similar across the groups, but the cognitive profiles were different.

Normalizing the pain was an emotional endeavor for the adolescents; occasionally the adolescents in maintenance category felt anger or sadness at having to adjust for the pain but all had managed to normalize the pain. In the management category, the adolescents clearly define the pain episodes, thereby distinguishing the moments when they are not in pain and when they are. Therefore, this group of adolescents reported negative emotions toward the pain because the pain existed, interfered with their lives, and would not allow them to feel normal like their peers. These patterns suggest that there are links between the coping goals and behaviors and their emotional representation of pain. Furthermore, these data signified that the emotional pathways were critical to an adolescent's coping behaviors. The way the adolescents perceived their pain was rooted in their emotional profiles. One group of adolescents viewed their pain as a normal occurrence and a challenge they adjusted and adapted to. The other group viewed their pain as a threat and dealt with the pain as episodic. In previous work into adolescents' journey to adjustment, their mechanisms to normalize JIA was through resistance and acceptance.^29^ Our findings as those of earlier research highlight how these conflicts between adjusting to a long‐term condition (aiming to maintain normality) and dealing with pain that can be all‐consuming. Such conflicts have also been found in other long‐term conditions such as eczema, where young people struggle with visible and invisible symptoms that create different psychosocial needs that are in conflict. These include the need to have their experiences of illness acknowledged and to be taken seriously/understood alongside the need to be seen as normal.^30^ These internal psychological conflicts create more negative emotional representations as highlighted in the current study. A recognition of these potentially conflicting goals and needs can inform support for these populations.

Research into pain coping has provided mixed results on the advantages of an individual focusing on controlling pain. Earlier approaches have classified coping as either adaptive or maladaptive,^31^, ^32^ but these classifications do not allow examination of the underlying processes and pathways that drive coping. In contrast, the CS‐SRM demonstrates how and why the appraisal of a threat provides direct motivation for the coping behavior.^15^

The literature has provided evidence for the same coping behavior to have both beneficial and adverse effects on pain. The current analysis found that although the adolescents had the same subordinate coping goal to preserve their social identity of normality, the coping behaviors on pursuing the goal can differ and two categories of imminent coping goals emerged. Using these categories to explore coping behavior showed the differences in what the adolescents and their parents focus on and use to motivate their behavior. This further supports exploring coping with chronic pain using a motivational perspective.^4^

Social comparison provides motivation in that it establishes a normative standard to aim for and from which they can develop their own personal goal. Social comparison facilitates self‐evaluation and drives goal formations.^33^ The assimilation and accommodation of coping as proposed by the dual‐process framework^20^ has been previously adapted successfully for chronic pain.^34^ In the current study, the adolescents' attempts to manage pain (assimilation), or normalizing pain (accommodation), enabled them to achieve some of those life goals that are viewed as expected for their age group. These overall attempts also coincide with functional coping literature with two classes of functional responses..^35^ By using this coping framework of functional behaviors and motivations, coping behaviors are appraised as either adaptive or maladaptive depending on the individual's goals. While the CS‐SRM framework allows for further examination of the underlying motivations to the coping goals and to the coping means.

Previous research has provided evidence that the meaning of pain is pivotal in reported pain intensity.^16^ In this current qualitative study, the adolescents' attached meaning to their pain is linked to the development of their self‐concept. There were two types of pain representations; either that pain is a threat, or it is a challenge to achieving their overall goal. These pain representations are driven by meanings designated by the adolescents.

Pain perceptions are influenced by the extent to which they view having JIA as a threat to a positive sense of identity. Adolescents could either develop a coherent sense of self in which JIA is just one aspect, or they develop alternative accounts of themselves whereby they contrast an identity with JIA with the one without JIA. In other conditions, it has been found that the degree to which individuals incorporate their illness into their sense of self predicts outcomes or severity. A study of adolescents with uncomplicated epilepsy found that a higher sense of coherence led to lower self‐reported illness severity.^36^ Similar results were found in a study where those adolescents who integrated their Type 1 diabetes with their sense of self (higher sense of coherence) had higher self‐esteem. They also reported better self‐management and fewer emotional and social problems.^37^

In the current study, the group of adolescents in the *maintenance* category associated the meaning of pain to a normal occurrence and integrated the chronic condition in their self‐concept. The *maintenance* category group also reported less pain intensity and higher function. In comparison, the group of adolescents in the *management* category associated their pain with episodes of flare‐ups. This association led to the adolescents in the *management* category to treat and perceive their condition as a series of acute pain episodes rather than a chronic pain. This can be mapped back to the underlying beliefs about the nature of the JIA where adolescents either understand JIA as a relapsing‐remitting condition or they are experiencing reoccurrences of the condition. However, further evidence of these underlying beliefs is required. Consequently, these adolescents had two conflicting identities and had higher emotional distress as well as reporting higher pain severity and lower function. Therefore, for these adolescents, they had a lower sense of coherence and possibly lower self‐worth. Associations between self‐perceptions and pain and function have been previously reported^38^ where higher pain was reported in those adolescents with lower self‐worth.

Although there were no differences in the demographic characteristics across the adolescents' groups the adolescents in the *management* category reported more pain at the point of data collection. These results suggest that what differentiates the two groups are not cognitive developmental issues, or the duration of the disease, but rather the perception of function and pain intensity. This observation has two possible interpretations. Either that the adolescents in the *management* category are initially motivated to manage the pain because they are experiencing more disability and higher pain than the adolescents in the *maintenance* category, or it could be they are feeling higher pain intensity as a result of their coping behaviors. Longitudinal research designs are required to address this question.

Furthermore, due to the nature of the interviews, the direction of the parental influence cannot be determined. The relationship between the adolescents' coping and parents' pain beliefs may be bi‐directional. One limitation of this work is that the connection between the two is not certain and further evidence of this connection would be required. The coping goals that the child engages in may also be influencing the parental beliefs about pain. Specifically influencing the parents' beliefs underlying the domains of control (personal and treatment), consequences, and timeline (chronicity and cyclical).

The choice of coping goals may directly result from the parent's perceptions and the child's emotional representations or it may be that we captured views and emotions resulting from engaging in the specific coping goal. Despite this, the patterns found within the two groups of coping styles suggest that these behaviors result from a family dynamic rather than characteristics of the young person. This supports the previous research in establishing the impact of the parental role.^7^, ^39^ This would further suggest that interventions designed for families to cope with pain would be most beneficial. Coping goals could be a route for such interventions if we can assess goals systematically.

Despite these limitations, one advantage of this current study is that it provides evidence that the adolescents' illness self‐concepts are related to their parents' cognitions. Some research has established a hypothesis that a parent's response to their child's pain impacts how that child copes.^10^, ^40^ Parental cognitions such as catastrophizing thoughts have been found to influence how the parent responds and what behavior the parent encourages.^13^ This current qualitative study demonstrated that there is a more complex relationship between parental beliefs and the type of coping goal that is encouraged and employed. Strength of the present study is the analysis of the matched parent‐child accounts of their pain beliefs and emotional representations through coping goals categories. This current study suggests that there is a direct link between the parents' beliefs, the parents' behaviors, and their children's coping behaviors. For this reason, this study suggests that there is a need to explore the parent and child dyad's pain beliefs simultaneously and within a CS‐SRM framework.

The patterns found in the parental beliefs and emotions suggested that the context of adolescents' coping behaviors should also be examined, considering the role of parental beliefs in the relationship between their child's pain experience and their child's coping goal. An early study in children with JIA found that greater emotional distress of the mother was related to higher levels of child‐reported pain.^41^ Further work into the parental role within a CS‐SRM framework can not only inform us how the relationship works but also proposes that for adolescents, the CS‐SRM includes a direct relationship between the parental beliefs and the coping behaviors. This emphasizes the need to include families' emotions, cognitions, and behaviors when examining pain. Further work into family communication about pain and family dynamics impact on coping with pain is required.

## 5 CONCLUSIONS

The CS‐SRM was used to understand the mechanisms and the extent to which parents influence the coping responses of adolescents to pain associated with JIA. This is especially important when examining pain, pain severity, and coping. The current study also highlighted the importance of the development of self and social identity for adolescents with a long‐term condition. Utilizing this theoretical framework to explore the motivation of coping identified these developmental milestones and provided targets for interventions in pain management.
